# Hospital and Institutional News

**Published:** 1919-06-21

**Authors:** 


### HOSPITAL AND INSTITUTIONAL NEWS.

### THE LATE COLONEL E. M. BRUCE VAUGHAN, V.D.

ALL connected with King Edward VII.'s Hospital and the Welsh National Medical School, Cardiff, have suffered a grievous loss through the death of Colonel Bruce Vaughan at the comparatively early age of sixty-two years. He died at Cardiff on Friday, 13th inst., having been in failing health for several months. An architect by profession, Colonel Bruce Vaughan specialised in ecclesiastical architecture, and throughout South Wales—particularly in Glamorganshire, Monmouthshire, and Carmarthenshire—the *Western Mail* testifies, there stand to his credit "modern edifices, renovated churches, internal adornments in the form of rood screens, reredos examples, and other things which are strikingly beautiful in design and execution." When Colonel Bruce Vaughan joined the Committee of King Edward VII.'s Hospital, Cardiff, in 1900, fifty of its available beds were closed down, and the annual expenditure exceeded the hospital income. He began his work for this hospital so quietly that no one dreamt that he would prove an active force in hospital reorganisation, and such suggestions as he made were generally regarded as not coming within the sphere of practical policy. In these early days for a few years Colonel Vaughan was evolving a programme of development and progress which, the historian records, "had he announced in its entirety, the Committee of the Cardiff Infirmary would have regarded him as a dangerous enthusiast." Colonel Vaughan had set his heart to render the existing buildings efficient, to increase their number and character considerably, and to provide the institution with an additional income to meet the increased costs, coupled with greater efficiency in administration. Beyond all this he had resolved to give his life to secure an important status for the hospital in association with the foundation and development of a medical school in connection with Cardiff University. With the co-operation of Mr. Thomas Andrews, a generous and public-spirited Mayor of Cardiff, and backed by the *Western Mail*, £12,568 was raised and the debt liquidated. Further benefit was conferred on King Edward VII.'s Hospital by directing public attention and sympathy to an especial degree and in an attractive manner. By steady, persistent, continuous work and devotion up to the time of his death Colonel Bruce Vaughan had succeeded in accomplishing nearly all the great things which he had it in his heart to attempt for Cardiff and its people when he joined the Committee of this hospital in 1900. In this way Colonel Bruce Vaughan did a wonderful and most excellent work for Wales and the hospitals within that principality in the relatively short period of less than twenty years. He put his whole mind and energies into the accomplishment of the task he had set himself. He had no ambitions for himself, and he was devoted heart and soul to the hospital of his choice, its Medical School, and everything connected with the prosperity and advancement of both. Colonel Vaughan was deservedly honoured by Welshmen of all shades of opinion. His death has caused universal grief, especially in Cardiff, where he was honoured and beloved by all classes of the community.

### A RECORD OF PROGRESS AND ACHIEVEMENT.

SOME idea of the work he did for the hospital and the Welsh Medical School must here follow. Through Colonel Bruce Vaughan's appeal 350 extra beds have been opened at the hospital, making provision for 6,000 additional patients per annum. The hospital income was increased by £20,000 a year, but the expenditure also increased by £27,000 a year. Since 1900 building extensions have been carried out at a cost of over £120,000. Colonel Vaughan was mainly responsible for securing financial assistance of something like half a million sterling. He further secured the linking up of the hospital with Cardiff University College, and obtained large sums to bring this scheme into actual existence, one gentleman, Sir W. J. Thomas, contributing no less than £100,000. To-day a large portion of the Medical School has been erected, and another wing is in course of construction, with the full consent and approval of the treasurer. Few men have rendered such invaluable service to the community amongst whom Colonel Vaughan's lot was cast, and he has proved to be an inspiring influence for good and an exemplar who, we hope, will be followed by many imitators, for the lasting benefit of the people of Wales.

### AN APPRECIATION BY A FELLOW-WORKER.

"I HAVE not the heart to write at the moment. Colonel Vaughan's death is a great grief—a personal loss to all of us, and his personality at the hospital is irreplacable. What was the cause of his wonderful success? I believe myself the motive of all his work was essentially religious. He had no special devotional gift, but he believed he was doing work for God at the hospital and in connection with the Medical School, and he was right, for God has abundantly blessed all his efforts. Then he had the crusading spirit. If he felt a thing ought to be done, he let nothing stand in his way. Persistently, in season and out of season, he hammered away until he achieved his end. He had a marvellous vision of what should be done. He did not think only for to-day or for to-morrow, but for very many years to come, and the greatness of his work will probably be more fully realised a decade hence. Then, again, he had a passion to help sick and suffering people. They made an instant appeal to him, and he rested neither day nor night if he could do anything to help them. How susceptible he was to the cry of suffering! With all his energy and driving force and ruthless progress to the goal before him, how hopelessly human he was after all—a strange mixture of unbending resolve and tender solicitude. A feature of his character was his essential disinterestedness. Superficially he was susceptible to praise and greatly helped by encouragement, but underneath it all he was a humble man, always content to sacrifice himself for the cause, always recognising that the cause was greater than he, and must go on. So, if he could speak now (and he does speak), he would cry to us all: 'Carry on, carry the torch that I have lighted, that the dark places, where disease lies lurking, may be illumined—that science and pity may bring God's gift of healing to more and more and more, especially in this great industrial district where my life has been spent'—and where, we may add, his memory will be revered for generations. As for his monument, it is the hospital—like Wren's, 'Circumspice.' As for himself, it is already written on the tablet on the 'Bruce Vaughan' Wing—'He whose care is for others, forgets to be afraid.' And long before that it was also written—'Yea, though I walk through the valley of the shadow of death, I will fear no evil; for Thou art with me; Thy rod and Thy staff comfort me.' Dear Colonel, Requiescat in Pace."

### "THE TIMES" AND MEDICAL ADVERTISEMENTS

In The Hospital for June 7, 1919, p. 236, exception was taken to an advertisement which had appeared in the *Times* on June 4, on the grounds that in offering to "cure" cases of cancer without operation the advertiser was misguided, and that the insertion of his offer was against the public interest. The advertisement in question had not been approved by the medical expert of the *Times*. The associate manager of the *Times* has courteously informed us that in future no advertisements of a similar nature will be accepted, a concession to the public interests which is just what would be expected from the management of our greatest newspaper. This is an example worthy of imitation by the rest of the Press. We are also informed that the advertiser in question is a registered practitioner, and we therefore repeat our invitation to the General Medical Council, within whose jurisdiction the matter appears to lie, to consider their attitude.

### A GIFT TO CAMBRIDGE.

For the building and equipment of a new nursing hostel in Cambridge £25,000 has been offered by Mr. C. Morland Agnew, M.A., of Trinity College, as a token of his gratitude for the recovery of Mrs. Agnew, who underwent a successful operation at the present nursing hostel. The gift has been accepted, and each college is to be asked to contribute towards a guarantee fund of not less than £750 for the expenses of the first year. Many similar hostels throughout the country are the pressing need of the moment. We hope gratitude will bring many others to follow so excellent an example.

### INFANT WELFARE AT GUY'S.

We understand that the Governors of Guy's Hospital have accepted a scheme for the organisation of an extensive children's department, and that the constructional work concerned is by this time well in hand. Apart from a children's ward of about fifty cots run in conjunction with a lying-in ward of twenty beds, the hospital will in future embody a complete infant-welfare centre which will undertake not only the supervision of children, but the ante-natal care of between 2,000 and 3,000 expectant mothers every year. Areas of Bermondsey, Southwark and Lambeth will have the additional advantage of twelve other centres, which will continue to act as in the past but with the valued lead and co-operation of the new organisation at Guy's. The hospital out-patient department will place at the disposal of the centres invaluable advantages of both diagnosis and treatment, and to us so well co-ordinated does the scheme appear, that the much-needed opportunity should be obtained to study and advance that most important aspect of the children's health problem—namely, prevention. Centralisation of this type brings obvious advantages both for the teaching of our future practitioners and bringing the true requirements of child-welfare work before the doctors of to-day. But even more immediately important, it will produce active, reliable guidance to the people of this country at a period when it is most urgently needed. We have often urged the necessity for closer and more extensive co-operation between the medical organisations of our great hospitals and the lesser welfare centres, and this development at Guy's receives our sincere commendations and wishes for success.

### FUTURE OF MEDICINAL MEN.

Speaking at Exeter at the annual meeting of the South-Western Branch of the B.M.A. Dr. Alfred Cox made an outline of the possibilities of the future for the medical profession, and spoken upon such occasion and following a number of both opposing and parallel forecasts which have lately appeared in the Press. Some of his suggestions may well be noted. Now that the Insurance organisation was taken over by the Ministry of Health reconstruction came under consideration, and the first step would be the creation of a new local health authority; and though it was important that the views of the profession should be fully represented during this development, it was not wise to allow the laity to come to consider the profession as a lot of truculent blackguards out for what they could get. On the new local authority there should be directly elected medical representatives. The Poor Law medical service would be merged into the new, greater service, and the question remained as to exactly who among the people should be catered for. He was very much opposed to a full-time medical service, particularly if it prevented the public from the right to select their own doctors. For the public benefit a part-time service should be adopted in which greater co-operation gave greater leisure, but at the same time the opportunity of closer supervision and development of a higher general standard of work. Extension of a State service might limit the chances of making big incomes by men of special ability, but the country practitioners' position would be relatively improved. If something were not done the doctor in rural England would be a thing of the past. Adequate remuneration must be obtained for him, as little interference as possible to come from lay people, and a service the conditions of which allowed him to do his best work. Dr. Cox appears to definitely favour the part-time service, but under very revised conditions after the former panel arrangements. To those who are watching the signs of the times, we think this speech is yet another which may be read with interest.

### FURTHER SMALLPOX EXTENSION.

It is reported that fears are entertained of an extension of smallpox in Glasgow. A seaman on holiday in Dundee after being demobilised from the Navy was aware of mild symptoms on May 21, but owing to the fact that the rash was disguised in character, he travelled to Govan by train, the condition being unsuspected as smallpox. The above is an example of how easily the disease may pass unrecognised by the modern medical man who may rarely have seen a case at all, but also, if we trace this particular instance further, the extensive danger coming from such infections in partially-vaccinated people becomes obvious. This particular man was employed in the Clyde yards from June 4 to 10, and other members of his family worked in various other areas. Two of them were at school. He arrived home about May 24, and since then his mother and sister have both developed smallpox. Of the known contacts, one developed the disease about the same time, and considering the very large number of people who have been in close proximity to one or other of the members of this family, it is not surprising that fears of an outbreak are entertained. Vaccination is now being offered locally to all possible contacts, and this is satisfactory up to a point, but as we have previously urged, it is prevention we need and local vaccination campaigns *before* such dangerous cases can appear in the districts concerned.

### THE DRUG HABIT.

The activity of American authorities in dealing with the drug question is both remarkable and far-reaching. In the New York State letters have been sent to mayors and health officers throughout this area asking them immediately to establish local clinics for the special treatment of those people addicted to the taking of drugs. Several conferences have been held to formulate plans to check the sale of these substances and to impose penalties upon the medical profession in instances of irregularity in issuing prescriptions. Both those objects are now in a large measure achieved, and considerable control obtained. Also the reflex effect of the prohibition order is both realised and expected. It will be a matter of considerable interest to learn facts of the damage to health, natural and individual, which the drug habit can produce, when this activity in the States has run long enough to give more detailed accounts of results and statistics.

### WOUNDED FROM ARCHANGEL.

Last week there disembarked at Leith from the hospital ship *Kalyan*, formerly a P. & O. liner, about 600 British and American soldiers from the North Russian war zone. Used meanwhile as a military hospital, the vessel has been at Archangel since last October, being icebound throughout the winter. News comes of the excellent work which the American Red Cross has done in this area both among their own troops and the natives. According to the *Times'* correspondent, they brought over nearly £400,000 of supplies and foodstuffs, and are at present organising further extensive relief measures in this particularly desolate area. Apparently, in spite of endless difficulties, the medical organisation and preventive work has been excellently carried out among the Allied forces, and from the sick and convalescent now arrived one gathers the satisfaction that war's lessons have been applied, and their stern value appreciated, even if this little party carry to us an abrupt reminder that peace has not yet descended upon all the world.

### THE LISTER INSTITUTE.

Recently has been issued the annual report of the Lister Institute of Preventive Medicine, and its appearance recalls the fact that we have in this body a great national asset, provided that continued support is forthcoming. The name is descriptive of its main object, and Sir David Bruce, the president, is one of the most famous of those who have sought and found many root causes of disease. To recount but a few of the Institute's accomplishments, we may mention its valuable work in the mass production of anti-tetanus toxin at the outbreak of war, the initiative and flexibility which allowed the lives of thousands to be saved. Also the work of the Trench Fever Committee was greatly helped by the Institute's investigations; while the solutions of problems of food, food poisoning, and accessory food factors were influenced and forwarded to an invaluable extent. In hardly any field of military operations has the effect of this work not been felt, and, apart from past achievement, such knowledge gives a magnificent share to the excellent foundations upon which we hope to build up our national health work in future. It is stated that a hospital is to be organised in connection with the Lister in realisation of the value of complete co-ordination of chemical and laboratory investigation.

### A HEALTH CONFERENCE.

On Wednesday, June 25, until the following Saturday, in London will be held, under the auspices of the Royal Institute of Public Health, a conference to consider problems of reconstruction in regard to public health under the following heads: The Work of the Ministry of Health, the Prevention and Arrest of Venereal Disease, Housing in Relation to National Health, Maternity and Child Welfare, and the Tuberculosis Problem and After-War Conditions. The inaugural meeting will take place at the Mansion House, under the presidency of Lord Leverhulme, and the sessions of the conference will be held in the Council Chamber at Guildhall on Thursday, Friday, and Saturday. One looks forward to some highly interesting and valued discussions on these matters, which are at the moment of outstanding importance.

### UNIVERSITY COLLEGE HOSPITAL.

It is announced that a war memorial scheme for this hospital has now been decided upon by a representative committee under the patronage of Lord Rosebery, and an appeal for a sum of £30,000 is now being issued to all old students of the College whose addresses are known. The scheme includes the following arrangements:—A war memorial album to contain the records of the academic and Service careers of the 268 men who have fallen; memorial tablets recording their names, and scholarships for the sons and daughters of the fallen; a Great Hall for the use of the College and Medical School and the endowment of University College Hall, Ealing. Captain Wedgwood Benn, M.P., is Hon. Treasurer, and donations should be sent to him at University College.

### APPARENT EPILEPSY.

A remarkable case of apparent epilepsy, which was in reality due to a bullet lodged in the ear of the young sufferer, is reported from Midhurst, Sussex, the patient being a twelve-year-old boy, the son of a sapper in the Royal Engineers. Some three months ago the boy's mother noticed that he had a wound in the right ear, for which he was quite unable to account. Deafness developed, and about six weeks later the child began to suffer from violent fits, which occurred at regular hourly intervals. Dr. B. E. G. Bailey was called in, and the boy was removed to the National Hospital for Epileptics at Bloomsbury. On examination there it was discovered that a small bullet had penetrated the right ear. With the removal of the missile the fits ceased, and young Verrall has now almost completely recovered his normal health. The boy is unable to give any explanation as to how the bullet got in his ear, and the matter remains a complete mystery.

### MARRIAGE AND TUBERCULOSIS

The extent of comment upon a recent breach of promise case, in which the defence established the fact that the plaintiff suffered from tuberculosis, shows that much public interest was excited. Unfortunately, the jury failed to agree. Hard as such instances may be upon female plaintiffs, there is the larger eugenic consideration to be looked to, and if a verdict had been returned for the defendant undoubtedly something would have been done towards hastening the day when the law (or, as M. Brieux thinks, much preferably custom) will require a certificate of health from both parties to a marriage. In the case under notice several newspapers failed to reckon with the point that a woman, once tuberculous, however seemingly recovered, always labours under a strong risk of having her disease re-awakened to activity by the strain of childbirth. Although ex-sanatorium female patients have been known to bear offspring successfully, yet the chances are the other way, and such women should not look to matrimony unless their prospective husband knows how matters stand.

### THE GARRETT ANDERSON HOSPITAL

The attempt to endow the Elizabeth Garrett Anderson Hospital, in memory of its founder, which was started in 1916, is so nearly successful that we hope a special effort will be made to make it so. Of the £50,000 required a sum of £6,500 is wanted, and the women who have done so much must now complete their task. Probably no women's profession or school has been omitted from the ranks of the hospital's supporters, but time is short, for the fund will probably be closed in the autumn. We wonder whether domestic servants have been interested in the scheme. They have received signal advances in wages during the past few years, and, though they are difficult to approach *en masse*, they have their own insurance society, and are beginning to take a professional, or at least a corporate, view of themselves.

### A NEW "PART-PAY" HOSPITAL.

With the closing of the Freemasons' War Hospitals at Fulham Palace and in Fulham Road the latter is about to become a part-payment hospital and nursing home for Freemasons and their families. The building is that which used to be known as the Chelsea Hospital for Women, and is therefore better fitted than many have been for work of the kind.

### A TIMELY LEGACY.

It is good news that the Paddington Green Children's Hospital is receiving, under the will of the late Mr. Joseph Lee Thomas, who lived in its neighbourhood, a legacy of £25,000. It is rather remarkable because it adjusts the average, which had been seriously reduced during the past seven years. Even so the increased income of some £1,200 a year is only equal to about half the deficit incurred during the past twelve months, and we imagine that Mr. F. S. Cheer has held the post of secretary just long enough to appreciate the difficulties of financing a hospital in Paddington. It has friends in the Homburg family, and some friendly support from the Great Western Railway, but we doubt whether the district has been financially organised to the full. The present legacy is the more gratifying, and we hope that it may attract others.

### THIS WEEK'S DRUG MARKET.

Business has recovered with unusual speed, and is now as brisk as it was a fortnight before Whitsuntide. On the whole prices are much steadier, and such articles as are still moving downwards in value are for the most part moving slowly. There has been a further advance in the price of camphor, and the general opinion seems to be that we shall see still higher prices before long. Menthol is still attracting attention, and the value is well maintained. American oil of peppermint, on the other hand, has an easier price tendency, and there is plenty of room for a downward movement. Star aniseed oil is in more inquiry, and prices are steadier. Cocaine moves off slowly, and the price is not firmly maintained. Among synthetic products, antifebrin, phenacetin, phenazone, salicylic acid, and sodium salicylate have still a downward price tendency, but aspirin seems to have reached a rather more stable level.

